# Associations between echocardiographic manifestations and bacterial species in patients with infective endocarditis: a cohort study

**DOI:** 10.1186/s12879-019-4682-z

**Published:** 2019-12-16

**Authors:** A. Damlin, K. Westling, E. Maret, C. Stålsby Lundborg, K. Caidahl, M. J. Eriksson

**Affiliations:** 1 Department of Molecular Medicine and Surgery, Division of Clinical Physiology, Karolinska Institutet, (L1:00), SE-171 76 Stockholm, Sweden; 2 Department of Clinical Physiology, Karolinska University Hospital, A8:01, Eugeniavägen 3, SE-171 76 Stockholm, Sweden; 3 Global Health – Health Systems and Policy: Medicines, focusing antibiotics. Department of Global Public Health, Karolinska Institutet, SE-171 77 Stockholm, Sweden; 4 Department of Medicine Huddinge, Division of Infectious Diseases and Dermatology, Karolinska Institutet, SE-141 86 Stockholm, Sweden; 5 Department of Infectious Diseases, Karolinska University Hospital Huddinge, SE-141 86 Stockholm, Sweden; 6 Institute of Medicine, Sahlgrenska Academy, University of Gothenburg, SE-413 45 Gothenburg, Sweden; 7 Region Västra Götaland, Department of Clinical Physiology, Sahlgrenska University Hospital, SE-413 45 Gothenburg, Sweden

**Keywords:** Infective endocarditis, Echocardiography, Heart valves, Diagnostics, Cardiac imaging

## Abstract

**Background:**

The diagnosis of infective endocarditis (IE) is based on microbiological analyses and diagnostic imaging of cardiac manifestations. Echocardiography (ECHO) is preferred for visualization of IE-induced cardiac manifestations. We investigated associations between bacterial infections and IE manifestations diagnosed by ECHO.

**Methods:**

In this cohort study, data from patients aged 18 years or above, with definite IE admitted at the Karolinska University Hospital between 2008 and 2017 were obtained from Swedish National Registry of Endocarditis. Bacteria registered as pathogen were primarily selected from positive blood culture and for patients with negative blood culture, bacteria found in culture or PCR from postoperative material was registered as pathogen. Patients with negative results from culture or PCR, and patients who did not undergo ECHO during hospital stay, were excluded. IE manifestations diagnosed by ECHO were obtained from the registry. Chi-squared test and two-sided Fisher’s exact test was used for comparisons between categorical variables, and student’s *t* test was used for continuous numerical variables. Multivariable analyses were performed using logistic regression. Secular trend analyses were performed using linear regression. Associations and the strength between the variables were estimated using odds ratios (ORs) with 95% confidence intervals (CIs). *P* < 0.05 was considered significant.

**Results:**

The most common bacteria were *Staphylococcus aureus* (*n* = 239, 49%) and viridans group streptococci (*n* = 102, 21%). The most common manifestations were vegetation in the mitral (*n* = 195, 40%), aortic (*n* = 190, 39%), and tricuspid valves (*n* = 108, 22%). Associations were seen between aortic valve vegetations and *Enterococcus faecalis* among patients with native aortic valves, between mitral valve vegetations and streptococci of group B or viridans group, between tricuspid valve vegetations and *S. aureus* among patients with intravenous drug abuse, and between perivalvular abscesses as well as cardiovascular implantable electronic device (CIED)-associated IE and coagulase negative staphylococci (all *P* < 0.05).

**Conclusions:**

Associations were found between certain bacterial species and specific ECHO manifestations. Our study contributes to a better understanding of IE manifestations and their underlying bacterial etiology, which pathogens can cause severe infections and might require close follow-up and surgical treatment.

## Background

Infective endocarditis (IE) is a serious infectious condition causing heart valve destruction, perivalvular abscesses, aneurysms, and fistulas, and can be lethal [1–3]. The diagnosis of IE is based on microbiological analyses and diagnostic imaging of cardiac manifestations. Echocardiography (ECHO) is the recommended imaging method for visualization of manifestations of IE, such as vegetations, morphological valve abnormalities/dehiscence, septal defects or fistula formation, and for the evaluation of hemodynamic consequences [4]. ECHO can be performed as transesophageal (TEE) or transthoracic (TTE) examinations [2]. Because TEE provides better image quality and has higher sensitivity and specificity for IE than TTE, it is recommended for all cases of prosthetic valves and intracardiac devices, and if a prior TTE had been negative but the clinical suspicion of IE is high. TEE should also be performed if the TTE is of low quality and is positive for IE, to rule out the possibility of local complications [1, 2].

Because bacteremia is common among patients with IE, microbiological analysis is an important step in the diagnosis of those with suspected IE. If the patients undergo cardiac surgery for IE, biological material can be taken directly from the focus of infection, for microbiological analysis with culture and 16S rRNA sequencing, which has a value especially for patients with blood culture-negative endocarditis [5]. This is recommended by the European guidelines for management of IE to optimize the choice of antibiotic treatment [2]. However, a positive culture confirming bacteremia is not sufficient to establish the diagnosis of IE [2, 6]. Further diagnostic criteria should be fulfilled, for example, one or more infectious manifestations verified by ECHO [2, 7].

Recommendations for the treatment of IE depend on the results from blood cultures, if the patients have native or prosthetic valves or cardiovascular implantable electronic devices (CIEDs), and are based on current evidence status [8]. Antibiotic treatment is recommended in clinically suspected cases of IE and if IE manifestations are present on ECHO but the blood cultures are negative [2, 9]. Although identification of causative bacteria and IE specific cardiac manifestations are crucial for the diagnosis and treatment of IE, the studies on associations between microbiological data and ECHO findings are scarce and the published results diverging [10–12]. Therefore, our aim was to analyze whether there were relationships between the bacteria found in blood culture or from sites of infection found during cardiac surgery and specific IE manifestations detected by ECHO in a cohort of IE patients. The secondary aim was to analyze bacterial species and ECHO findings among patients in the cohort that underwent surgical treatment for IE, had prosthetic valves and patients with intravenous (IV) drug abuse. The third aim was to study in-hospital mortality in the included patients.

## Methods

### Patients, study design and data selection

We performed a retrospective cohort study based on data from the Swedish National Registry of Infective Endocarditis (SRIE), which was established in 1995. Online reporting by Internet started in 2008 and all Swedish departments for infectious diseases report data from patients treated for possible or definite IE (ICD codes I33.0, I33.9, I38.9, and I39.8). The coverage for patients with IE is estimated to be 70–80% [13]. The SRIE includes variables such as gender, age, blood cultures, microbiological findings from samples taken during cardiac surgery, comorbidities and structural heart disease such as rheumatic heart disease, congenital heart disease, known valvular disease and heart failure, ECHO findings, as well as risk factors such as IV drug abuse, prosthetic valves (time for primary prosthetic valve implantation is not available). Furthermore, the SRIE provides data on antibiotics prescribed, duration of antibiotic treatment, and in-hospital mortality (defined as death during hospital stay). For this study, data from the SRIE were analyzed to identify a cohort of patients with IE treated at the Karolinska University Hospital (KUH), Stockholm, Sweden, a tertiary referral center for IE. All patients aged ≥18 years admitted to the Department for Infectious Diseases at KUH with definite IE between January 1, 2008 and December 31, 2017, with positive blood culture or positive culture or polymerase chain reaction (PCR) amplification from material sampled during cardiac surgery were obtained from the SRIE, giving a total of 513 patients. The ECHO investigations (TTE and TEE) were performed at the Department of Clinical Physiology in accordance with existing guidelines [2].

### Data management

Patients that did not undergo ECHO during hospital stay (21 patients), were excluded. Bacteria registered as possible infectious pathogens were primarily selected from positive blood culture results in 490 patients. For an additional two patients having negative blood cultures, the registered pathogens were found in culture or by PCR amplification from material sampled during cardiac surgery.

Thus, 492 patients were included in the study. Among the patients with positive blood culture results, 19 were registered with more than one bacterial species, or bacteria plus fungi; for these patients, the first pathogen listed was chosen for the statistical analysis. Of all the included patients who had positive results from both blood culture and culture or PCR from heart valve material, all were positive for the same bacterium in blood culture as in culture or PCR amplification from heart valve material. Coagulase-negative staphylococci (CoNS) were grouped including *Staphylococcus lugdunensis* and *Staphylococcus epidermidis* and the following bacteria were grouped as HACEK: *Haemophilus* species, Aggregatibacter species, *Cardiobacterium hominis*, *Eikenella corrodens*, and *Kingella kingae*. The IE manifestations were detected during TEE or TTE. If more than one ECHO finding was registered for the same patient, all manifestations were analyzed both separately and together. Data from the patient cohort were analyzed for age, gender, history of any IV drug abuse, presence of prosthetic heart valves, and for in-hospital mortality.

### Statistical analyses

Analyses were performed using STATA software (version 15.1 Stata Corp., College Station, Texas, USA). Frequencies and percentages were calculated for categorical variables and numeric variables are presented as the mean and standard deviation (SD) or median and 25th and 75th percentiles, when appropriate. For comparisons between categorical variables, the chi-squared test was conducted for values ≥5 and the two-sided Fisher’s exact test for values < 5 and presented with odds ratio (OR) with 95% confidence intervals (CIs). Student’s *t* test was used for continuous numerical variables; two-sided and skewed variables were log-transformed before these analyses. Multivariable analyses were performed using logistic regression. Secular trend analyses were performed using linear regression and t was obtained as a measure for the slope. A positive t shows a positive trend and a negative t shows a negative trend, over the study period. *P* < 0.05 was considered significant.

## Results

### Clinical characteristics

In total, 492 patients with definite IE according to modified Duke’s criteria for IE, were included in our study [2, 14]. The clinical characteristics and predisposing factors of the included patients are presented in Table 1. There were no differences in mean age between male (56.8 years) and female patients (57.8, *P* = 0.58). The most common bacterial pathogens were *S. aureus*, present in 239 (49%) patients, followed by viridans group streptococci in 102 (21%) and *Enterococcus faecalis in* 50 (10%). There were no differences in etiology between male and female patients. In total, 435 (88%) of the patients underwent TEE, 270 (56%) underwent both TTE and TEE and 57 (12%) underwent TTE only. The most frequent ECHO manifestations were mitral valve vegetation (*n* = 195, 40%), aortic valve vegetation (*n* = 190, 39%), and tricuspid valve vegetation (*n* = 108, 22%). Among all patients, 409 (83%) had one IE manifestation, 83 (17%) had more than one IE manifestation detected by ECHO. The most commonly combined IE manifestations were aortic valve vegetation and mitral valve vegetation (35 patients) and aortic valve vegetation and abscess (26 patients). Total in-hospital mortality was 7% (33 patients).

**Table 1:** Descriptive data of IE-patients admitted to the Karolinska University Hospital (KUH) from 2008 to 2017

**All patients n (%)**	492 (100)
Women	161 (33)
Men	331 (67)
Age, mean (± SD); medians (25th and 75th percentiles)	57.1 (18.4); 57 (44.5, 72)
**Predisposing factors**	
History of IV drug abuse	156 (32)
Bicuspid aortic valve	18 (4)
vProsthetic valve	92 (19)
CIED	50 (10)
Rheumatic heart disease	2 (0)
Congenital heart disease	7 (1)
History of IE	78 (16)
Known valvular disease	75 (15)
Heart failure before or under IE treatment	60 (12)
**Clinical characteristics**	
Fever	432 (88)
Vascular phenomena	210 (43)
New heart murmur	77 (16)

Notes: vales are presented in n = number of patients and % of all patients in parenthesis. Abbreviations: CIED, cardiovascular implantable electronic device; IE, infective endocarditis; IV, intravenous; KUH, Karolinska University Hospital; SD, standard deviation

### Bacterial species and ECHO manifestations

Distributions and associations between etiology and IE manifestations detected by ECHO are presented in Table 2. *S. aureus* was associated with tricuspid valve vegetation but not associated with aortic valve vegetation (Table 2). There were associations between *S. aureus* and pulmonary valve vegetation, although few patients had pulmonary valve vegetation (*n* = 9, 2%). Significant associations were seen between CoNS and perivalvular abscess, and the incidence of patients with more than one IE manifestation. Further, patients with *E. faecalis* etiology were more likely to have aortic valve vegetation. Among patients with a native aortic valve, the presence of aortic valve vegetations was significantly associated to findings of *E. faecalis* (OR 3.64, 95% CI 1.65–8.24; *P* < 0.01) and closely significantly associated to CoNS (OR 2.83, 95% CI 0.91–9.14; *P* = 0.04). However, among patients with prosthetic aortic valve, there were no significant associations between aortic valve vegetations and verified circulating *E. faecalis* (OR 0.67, 95% CI 0.20–2.36; *P* = 0.47) or CoNs (OR 3.89, 95% CI 0.46–180.98; *P* = 0.18). Patients with group B streptococci or viridans group streptococci were more likely to have mitral valve vegetation. Patients with viridans group streptococci were less likely to have tricuspid valve vegetation. Patients with HACEK multiple infections were more likely to have CIED-associated IE.

**Table 2:** Distribution of IE-manifestations and bacterial species comprising 90% of the etiologies among patients with IE

	Aortic valve vegetation	Mitral valve vegetation	Tricuspid valve vegetation	Pulmonary valve vegetation	CIED-associated IE	Perivalvular abscess	Total, n (%)
All patients, n (%)	190 (39)	195 (40)	108 (22)	9 (2)	24 (5)	29 (6)	
*S. aureus*	**68 (14), 0.43, 0.29–0.63; < 0.01**	84 (17), 0.69, 0.47–1.01; 0.05	**88 (18), 6.79, 3.93–12.11; < 0.01**	**8 (2), 8.73, 1.15–388.69; 0.01**	11 (2), 0.89, 0.35–2.20; 0.78	11 (2), 0.63, 0.26–1.44; 0.24	239 (49)
CoNS	**16 (3), 3.38, 1.33–9.29; < 0.01**	6 (1), 0.49, 0.16–1.33; 0.13	2 (0), 0.31, 0.03–1.30; 0.13	0 (0)	**4 (1), 4.48, 1.01–15.15; 0.02**	**5 (1), 4.87, 1.30–15.01; < 0.01**	24 (5)
Viridans group streptococci	45 (9), 1.33, 0.84–2.12; 0.20	**51 (10), 1.70, 1.07–2.71; 0.02**	**5 (1), 0.14, 0.04–0.36; < 0.01**	0 (0)	**1 (0), 0.16, 0.00–1.00; 0.04**	6 (1), 1.00, 0.32–2.61; 1.00	102 (21)
Group B streptococci	4 (1), 0.91, 0.19–3.62; 1.00	**9 (2), 7.13, 1.44–68.33; < 0.01**	0 (0)	0 (0)	0 (0)	1 (0), 1.62, 0.04–12.11; 0.49	11 (2)
Group D streptococci	3 (1), 1.20, 0.17–7.15; 1.00	4 (1), 2.05, 0.34–14.14; 0.44	0 (0)	0 (0)	0 (0)	1 (0), 2.70, 0.06–23.37; 0.35	7 (1)
Group G streptococci	4 (1), 1.60, 0.29–8.70; 0.49	3 (1), 0.91, 0.14–4.73; 1.00	1 (0), 0.50, 0.01–3.99; 1.00	0 (0)	0 (0)	0 (0)	8 (2)
*E. faecalis*	**30 (6), 2.64, 1.40–5.08; < 0.01**	19 (4), 0.93, 0.48–1.75; 0.80	7 (1), 0.55, 0.20–1.28; 0.15	0 (0)	2 (0), 0.80, 0.09–3.41; 1.00	2 (0), 0.64, 0.07–2.69; 0.76	50 (10)
HACEK	6 (1), 1.06, 0.31–3.40; 0.91	3 (1), 0.37, 0.07–1.40; 0.18	2 (0), 0.54, 0.06–2.44; 0.54	1 (0) 4.19, 0.09–34.97; 0.25	**4 (1), 8.31, 1.76–31.08; < 0.01**	1 (0), 1.15, 0.03–8.07; 0.60	15 (3)

Notes: The table represents all included patients, with native and prosthetic valves analyzed together. Values are presented in n = number of patients, (% of patients), odds ratio, 95% confidence interval; *P-value.* Statistically significant associations (*P* < 0.05) are shown in bold. Abbreviations: CIED, cardiovascular implantable electronic device; CoNS, coagulase-negative staphylococci; E, enterococcus; HACEK, *Haemophilus* species, *Aggregatibacter* species, *Cardiobacterium hominis*, *Eikenella corrodens*, and *Kingella kingae*; IE, infective endocarditis; KUH, Karolinska University Hospital, S, staphylococcus

### Bacterial species and ECHO manifestations among patients with IV drug abuse and prosthetic heart valves

Patients with IV drug abuse were more likely to have an etiology of *S. aureus* infection but less likely to have CoNS or viridans group streptococcal-related IE (Table 3). Furthermore, such patients were more likely to have tricuspid valve IE and pulmonary valve IE but less likely to have aortic and mitral valve vegetations (Table 4). Of the 239 patients with *S. aureus*-linked IE, 122 had a history of IV drug abuse and 117 did not. The associations between *S. aureus* and tricuspid valve vegetation when adjusted for IV drug abuse and *S. aureus* IE were associated with tricuspid valve vegetation only among patients with IV drug abuse (OR 4.75, 95% CI 1.92–12.52; *P* < 0.01) but not among patients who did not have IV drug abuse (OR 1.96, 95% CI 0.74–5.16; *P* = 0.12).

**Table 3:** Distribution of bacterial etiologies, predisposing factors and outcomes among patients with IE at the KUH

	Prosthetic heart valve	IV drug abuse	> 1 ECHO manifestation	Surgical treatment for IE	In-hospital mortality
All patients, n (%)	92 (19)	156 (32)	83 (17)	139 (28)	33 (7)
*Staphylococcus aureus*	**28 (30), 0.39, 0.23–0.65; < 0.01**	**122 (78), 6.72, 4.24–10.76; < 0.01**	36 (43), 0.78, 0.47–1.28; 0.30	61 (44), 0.77, 0.51–1.16; 0.19	21 (64), 1.93, 0.88–4.42; 0.07
CoNS	8 (9), 2.29, 0.82–5.88; 0.06	**2 (1), 0.19, 0.02–0.77; 0.01**	**11 (13), 4.65, 1.80–11.71; < 0.01**	10 (7), 1.88, 0.73–4.67; 0.13	2 (6), 1.28, 0.14–5.62; 0.67
Viridans group streptococci	14 (15), 0.64, 0.32–1.20; 0.15	**8 (5), 0.14, 0.06–0.30; < 0.01**	14 (17), 0.74, 0.37–1.41; 0.34	34 (24), 1.36, 0.82–2.22; 0.20	6 (18), 0.84, 0.28–2.16; 0.71
Group B streptococci	1 (1), 0.43, 0.01–3.09; 0.70	0 (0)	2 (2), 1.10, 0.11–5.44; 1.00	3 (2), 0.95, 0.16–4.04; 1.00	1 (3), 1.38, 0.04–10.22;0.54
Group D streptococci	3 (3), 3.34, 0.48–20.04; 0.13	0 (0)	1 (1), 0.82, 0.02–6.89; 1.00	4 (3), 3.46, 0.57–23.84; 0.10	0 (0)
Group G streptococci	2 (2), 1.46, 0.14–8.33; 0.65	1 (1), 0.30, 0.01–2.40; 0.45	0 (0)	1 (1), 0.36, 0.01–2.83; 0.45	0 (0)
*Enterococcus faecalis*	**17 (18), 2.52, 1.25–4.93; < 0.01**	17 (11), 1.12, 0.57–2.16; 0.71	11 (13), 1.45, 0.64–3.05; 0.31	9 (6), 0.53, 0.22–1.14; 0.09	2 (6), 0.55, 0.06–2.29; 0.56
HACEK	5 (5), 2.24, 0.58–7.39; 0.17	0 (0)	4 (5), 1.83, 0.41–6.38; 0.30	**8 (6), 3.02, 0.93–9.96; 0.03**	1 (3), 0.99, 0.03–6.94; 1.00

Notes: Values are presented in n = number of patients, (% of patients), odds ratio, 95% confidence interval; *P-value.* Statistically significant associations (*P* < 0.05) are shown in bold. Abbreviations: CoNS, coagulase-negative staphylococci; ECHO, echocardiography; HACEK, *Haemophilus* species, *Aggregatibacter* species, *Cardiobacterium hominis*, *Eikenella corrodens*, and *Kingella kingae*; IE, infective endocarditis; IV, intravenous; KUH, Karolinska University Hospital

**Table 4:** Distribution of ECHO-manifestations, predisposing factors and outcomes among patients with IE at the KUH

	IV drug abuse	Surgical treatment for IE	In-hospital mortality
All patients, n (%)	156 (32)	139 (28)	33 (7)
Aortic valve vegetation	**31 (20), 0.28, 0.17–0.44; < 0.01**	**72 (52), 2.14, 1.41–3.25; < 0.01**	17 (52), 1.76, 0.81–3.82; 0.12
Mitral valve vegetation	**44 (28), 0.48, 0.31–0.74; < 0.01**	64 (46), 1.45, 0.95–2.19; 0.07	15 (45), 1.29, 0.59–2.79; 0.48
Tricuspid valve vegetation	**86 (55), 17.54, 9.99–31.33; < 0.01**	**19 (14), 0.47, 0.25–0.82; 0.01**	3 (9), 0.34, 0.06–1.12; 0.08
Pulmonary valve vegetation	**7 (4), 7.85, 1.46–77.92; < 0.01**	1 (1), 0.31, 0.01–2.37; 0.46	0 (0)
CIED IE	**1 (1), 0.09, 0.01–0.55; < 0.01**	**18 (13), 8.60, 3.16–26.96; < 0.01**	2 (6), 1.28, 0.14–5.61; 0.67
Perivalvular abscess	**4 (3), 0.33, 0.08–0.97; 0.04**	**17 (12), 3.96, 1.72–9.34; < 0.01**	**6 (18), 4.21, 1.29–11.81; < 0.01**
Prosthetic heart valve	**12 (8), 0.28, 0.13–0.53; < 0.01**	26 (19), 1.00, 0.58–1.69; 1.00	8 (24), 1.43, 0.54–3.41; 0.40

Notes: Values are presented in n = number of patients, (% of patients), odds ratio, 95% confidence interval; *P-value.* Statistically significant associations (*P* < 0.05) are shown in bold. Abbreviations: CIED, cardiovascular implantable electronic device; IE, infective endocarditis; IV, intravenous; KUH, Karolinska University Hospital

In our cohort, 92 patients (19%) had prosthetic heart valves, and 7 (8%) of these patients had two prosthetic heart valves. Such patients were more likely to have *E. faecalis*-linked IE, but they were less likely to have IE caused by *S. aureus*. Etiologies among patients with prosthetic heart valves are presented in Table 3. There was a significant association between the incidence of prosthetic heart valves and perivalvular abscesses (no specific locations were registered) (OR 17.64, 95% CI 6.89–50.34; *P* < 0.01). The described associations refer to the whole cohort of prosthetic IE in our study without differentiation between early and late onset prosthetic IE, which was not possible.

### Surgical treatment

In total, 139 (28%) patients were treated with cardiac surgery. There was no difference in age between patients who did or did not undergo surgical treatment for IE (surgery mean age was 55 years, non-surgery mean age was 57.9 years; *P* = 0.12). Of patients who underwent surgery, 26 had a prosthetic valve IE and 113 had a native valve IE. There was no difference in surgical treatment between these two groups (OR 1.00, 95% CI 0.58–1.69; *P* = 1.00). Patients with a history of IV drug abuse were treated less frequently with cardiac surgery than patients who did not have history of IV drug abuse (26 patients, 17%, OR 0.39, 95% CI 0.23–0.65; *P* < 0.01). Surgical treatment was more common among patients with aortic valve vegetation, perivalvular abscess, and CIED endocarditis and less common among patients with tricuspid valve vegetation (Table 4). However, a majority of the patients with tricuspid valve vegetation had history of IV drug abuse (*n* = 86, 80%). There were no significant differences in in-hospital mortality between patients who had surgical treatment for IE (*n* = 12, 2%) and those who did not (*n* = 21, 4%; OR 1.49; 95% CI 0.65–3.29; *P* = 0.28). In total, 26 patients had aortic root abscesses of whom 17 (65%) were treated with cardiac surgery; of these 17, three died during hospital stay. Among the remaining nine patients for whom surgery was not performed, two died during hospital stay. In patients presenting with an aortic root abscess, there was no significant association between surgical treatment and in-hospital mortality (OR 0.75, 95% CI 0.07–11.07; *P* = 1.00), nor between nonsurgical treatment and death (OR 1.33, 95% CI 0.09–4.58; *P* = 1.00).

### In-hospital mortality

The overall in-hospital mortality was 7% (33 patients). An association was seen between in-hospital mortality and an ECHO finding of a perivalvular abscess (*n* = 6, OR 4.21, 95% CI 1.29–11.81; *P* < 0.01, Table 4). Among the 26 patients with aortic root abscesses, 5 patients died during hospital stay, (19% in-hospital mortality). Among patients with *S. aureus*-linked IE, in-hospital mortality was more common among patients with left-sided valve IE (*n* = 14, 6%) compared with patients with right-sided valve IE (*n* = 1, < 1%; OR 9.63, 95% CI 1.40–412.09; *P* = 0.01). Among patients with *S. aureus*-linked IE, those with left-sided valve IE were significantly older than those with right-sided valve IE (mean age for right-sided valve IE was 38.2 years, mean age for left-sided valve IE was 60.3 years; *P* < 0.01). Further, IV drug abuse was more common among patients with right-sided valve IE, 72% (81 patients) of the 113 patients with right-sided valve IE had IV drug abuse.

Prosthetic valve IE was not associated with in-hospital mortality (OR 1.43, 95% CI 0.54–3.41; *P* = 0.40). There was a significant association between more than one IE manifestation on ECHO and in-hospital mortality (OR 2.69, 95% CI 1.12–6.10; *P* < 0.01).

### Gender differences

There were no significant differences in bacterial species between male and female patients, although male patients were more likely to have aortic valve vegetation than female patients (OR 1.77, 95% CI 1.16–2.71; *P* = 0.01). However, bicuspid aortic valves were closely significantly more common in male than in female patients (OR 4.04, 95% CI 0.93–36.56; *P* = 0.05). There were no differences between male and female patients in terms of history of IV drug abuse (OR 0.92, 95% CI 0.60–1.41; *P* = 0.69) or in the incidences of prosthetic heart valves (OR 1.07, 95% CI 0.64–1.81; *P* = 0.79), in-hospital mortality (OR 0.84, 95% CI 0.38–1.93; *P* = 0.64), or surgical treatment for IE (OR 1.35, 95% CI 0.86–2.14; *P* = 0.17).

### Differences over time

**Fig. 1:**
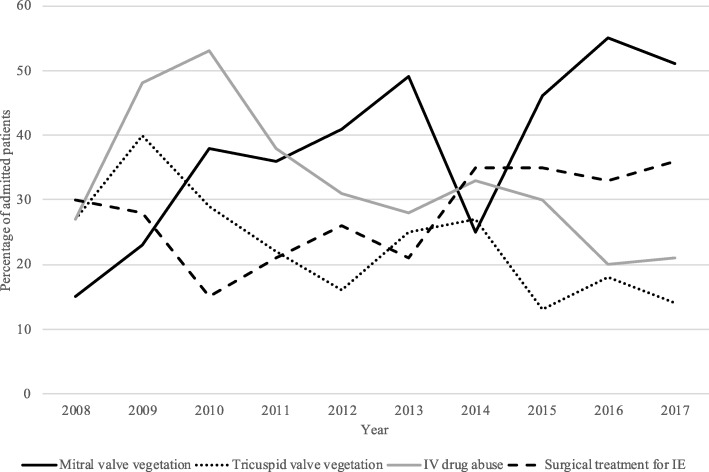
**(attached separately).** Trends of ECHO-manifestations, IV drug abuse and surgical treatment among patients with IE from 2008 to 2017. Notes: The y-axis presents the percentage of patients of all patients with IE during each year. Values for trend (t), followed by *p*-values are: mitral valve vegetation (t = 4.18; *P* < 0.01), tricuspid valve vegetation (t = − 3.01; P < 0.01), IV drug abuse (t = − 3.33; P < 0.01), surgical treatment for IE (T = 2.11; *P* = 0.04). Abbreviations: ECHO, echocardiography; IE, infective endocarditis; IV, intravenous

During study period, from 2008 to 2017, the incidence of mitral valve vegetations increased significantly (t = 1.27; *P* < 0.01) as did the incidence of surgical treatment of IE (t = 2.11; *P* = 0.04). Further, the incidence of tricuspid valve vegetations and IE-patients with IV drug abuse both decreased during the study period (tricuspid valve vegetation: t = − 3.01; *P* < 0.01; IV drug abuse: t = − 3.33; *P* < 0.01). The incidences of aortic or pulmonary valve vegetations, CIED-associated endocarditis or perivalvular abscess did not change during the study period, nor did the incidence of in-hospital mortality or prevalence of prosthetic valves among IE-patients. The secular trend analysis is presented in Table 5 and significant trends are presented in Fig. 1.

**Table 5:** Trends of ECHO-manifestations, predisposing factors and outcomes among patients with IE from 2008 to 2017

	t	p-value
Aortic valve vegetation	−1.27	0.21
Mitral valve vegetation	4.18	**< 0.01**
Tricuspid valve vegetation	−3.01	**< 0.01**
Pulmonary valve vegetation	0.05	0.96
CIED-associated IE	−0.29	0.77
Perivalvular abscess	−0.39	0.70
In-hospital mortality	0.16	0.87
IV drug abuse	−3.33	**< 0.01**
Prosthetic valve IE	0.82	0.41
Surgical treatment for IE	2.11	**0.04**

Notes: Value for trend (t), is obtained by linear regression. Statistically significant values (*P* < 0.05) are shown in bold. Abbreviations: CIED, cardiovascular implantable electronic device; ECHO, echocardiography; IE, infective endocarditis; IV, intravenous

## Discussion

To our knowledge, this is the first study showing significant associations between causative bacteria and IE manifestations detected by ECHO. In this cohort of 492 patients from the SRIE, we found that patients with *S. aureus* were more likely to have tricuspid valve vegetation but less likely to have aortic valve vegetation. Furthermore, *S. aureus*-linked IE was more common among patients with a history of IV drug abuse but less so among patients with prosthetic valves. Associations were found between CoNS and the presence of a perivalvular abscess, with more than one manifestation on ECHO and with CIED-associated IE, but CoNS were less common among patients with a history of IV drug abuse. Although it should be noted that there were relatively few patients with CoNS (*n* = 24, 5%). Among patients with a native aortic valve, findings of aortic valve vegetations were associated to presence of *E. faecalis*, and closely associated to CoNS. However, this was not the case with prosthetic aortic valves. Further, significant associations were seen between viridans group streptococci and group B streptococcal infections and mitral valve vegetations and between HACEK- and CIED-associated IE.

Associations between specific bacterial infections and IE manifestations detected by ECHO have been reported previously [10, 11]. Trifunovic et al. did not show any associations between specific IE manifestations and certain etiologies in their study of 246 patients with IE, even though their main findings were that *S. aureus* and gram-negative bacteria caused large vegetations, CoNS caused destructive leaflet lesions, CoNS and gram-negative bacteria caused perivalvular extension of the infective process, and that gram-negative bacteria were associated with multiple manifestations in the same patient [11]. Those results were consistent with our findings that CoNS infections were associated with perivalvular abscesses (extension of the infective process). Furthermore, we found that *S. aureus* infections were associated with tricuspid valve IE, which is supported by Bonetti et al. in their study of 274 patients with IE [10].

### Microbiological findings

In our analysis of the most common bacteria causing IE was *S. aureus*, present in 47% of all patients. These findings are in line with studies showing that *S. aureus* is the most common bacterium causing IE in industrialized countries [3, 12, 15, 16]. In this study, 51% of the patients with *S. aureus* etiology had IV drug abuse. However, when stratified for left-sided and right-sided IE, patients with *S. aureus* and left-sided IE were older and had significantly higher in-hospital mortality than those with *S. aureus* and right-sided IE. This stratified finding is more adherent to previous studies presenting that IE caused by *S. aureus* has high mortality rates compared to IE caused by other pathogens [3, 12, 15, 16]. In addition, we found that the presence of a perivalvular abscess, a serious IE manifestation of tissue destruction, was associated with in-hospital mortality. Similar results were reported by Lauridsen et al. who showed that perivalvular abscess and valve perforation independently predicted 1-year mortality in patients with left-sided native valve *S. aureus*-linked endocarditis [17].

Furthermore, in our study, *Streptococcus* species were also common (27%), as was seen in studies presenting a high and increasing prevalence of streptococci in general, but more specifically viridans group streptococci and *S. bovis* [18, 19].

### Surgery

In our study, 28% of patients underwent surgical treatment, slightly less than the 31% of Swedish patients with IE who underwent surgical treatment in 2017 [10]. This lower percentage in our cohort might be explained by the large proportion of patients with a history of IV drug abuse and right-sided IE, which were less frequently treated with cardiac surgery compared to the IE patients with no IV drug abuse. Patients with aortic valve vegetation, CIED-associated endocarditis, or a perivalvular abscess more often underwent surgical treatments. It has been argued whether *S. aureus* should be listed as an absolute indication for surgical treatment, as it often causes severe IE with the presence of emboli or abscesses, and/or severe valvular engagement, but the current recommendations promote individual evaluation of patients for decisions on using surgical treatments [6, 20, 21]. This individualized approach was adopted in decision-making at KUH.

### Prosthetic valves and CIED-linked IE

It has been reported that prosthetic valve IE and CIED-linked IE are becoming more frequent [18]. During the study period of this study, 2008–2017, there were no significant differences in the prevalence of CIED-associated IE and prosthetic valve IE, although it should be noted that there were only a few patients with CIED-linked IE and prosthetic valve IE. These cases can be challenging to diagnose, especially with TTE because of its relatively low resolution and shading from the prosthesis or chordae tendinae; thus, TEE is highly recommended [2, 18, 22]. The respective sensitivities for diagnosing native IE and prosthetic valve IE are approximately 96 and 70% with TTE and 96 and 92% for TEE, respectively [2]. In our study, associations were found between prosthetic valve IE and *S. aureus* and *E. faecalis, respectively.* The associations between the incidences of prosthetic valve IE and *E. faecalis* infection found in our study differ from previous reports. *S. aureus* and CoNS infections have been described as common etiological factors for prosthetic valve IE [19].

### IV drug abuse

KUH has a special ward for addicts with infections, with an uptake area covering Stockholm County Council (approximately 2.3 million inhabitants), which might contribute to the relatively high presence of IV drug abuse among endocarditis patients at KUH. Studies of patients with IE in the USA in 2012–2013 showed a lower incidence of IV drug abuse among IE patients (6.5–7.8% of the IE patients had IV drug abuse) than in our results (32% of the patients had a history of IV drug abuse), although recent US and European studies have noted that the presence of IV drug abuse is generally increasing among patients with IE [23, 24]. The high prevalence of IV drug abusers among the IE-patients have affected the association between *S. aureus* and tricuspid valve vegetation found in this study. When stratifying for IV drug abuse, we found that there were no association between *S. aureus* and tricuspid valve vegetation among patients with no IV drug abuse, but significant association was found among patients with IV drug abuse. *S. aureus* was the most common bacterial species among patients with IE and a history of IV drug abuse, which is consistent with previous studies [23–25].

### Gender differences

Although there were significantly more men than women in our study (~ 2:1), we found no gender differences in IE etiology, which is consistent with earlier studies in which the ratio of men to women was typically higher than 2:1 [26, 27]. Previous work has evaluated the differences in etiology and manifestations, but with ambiguous results. For instance, Aksoy et al. showed that women were more likely to have vegetations on CIED and men were more commonly infected with CoNS [26], while Sambola et al. showed that mitral valve IE and aortic valve IE were more common among men, but the etiology did not differ between genders [27]. In our study, men were more likely to have aortic valve IE, which is partly adherent to previous studies [27, 28].

The rates of in-hospital mortality and surgical treatment for IE in our study did not differ between men and women, which does not accord with previous reports indicating that women have higher mortality and receive surgical treatment to a lesser extent compared with men [10]. In our cohort, the in-hospital mortality rate was low (7%), which contrasts with previously reported rates of 15–20% [29, 30], but is more in line with a Swedish study presenting a 30-days crude mortality rate of 10.4% [3]. The low in-hospital mortality rate might be explained by the relatively low age of the patients (mean age 57.1 years) and high prevalence of right-sided, IV drug abuse-associated IE in our study.

### Diagnosis of IE manifestations

TEE has been reported to have high sensitivity for the diagnosis of IE, ranging between 90 and 100%, as well as a high negative predictive value of 86–97% [22, 31]. Moreover, it has been shown that ECHO findings did not differ between patients who underwent ECHO early (< 2 days) or late (≥2 days) after starting antibiotic treatment for IE [12]. We found IE manifestations most frequently on the mitral valve (*n* = 195, 40%), followed by the aortic valve (*n* = 190, 39%) and the tricuspid valve (*n* = 108, 22%). Our results resemble those of a study involving 68 autopsies of patients with IE, which reported that 35% of patients had mitral valve IE, 26% had aortic valve IE, and 5% had tricuspid valve IE [32]. Our higher prevalence of tricuspid valve IE may be explained by the high prevalence of IV drug abuse among the patients in our cohort. Our results are also consistent with a multicenter study of 1055 patients in Europe and the USA, showing that the most common ECHO manifestations among patients with IE were on the mitral valve followed by the aortic valve [33].

### Limitations

The SRIE includes variables on IE manifestation, such as vegetation localization, but not the size or numbers of vegetations which should have been valuable to present together with the results. The SRIE does include information about known valvular disease prior to IE diagnosis, but no detailed information about the type or severity of degenerative structural heart diseases, such as aortic stenosis is available. Further, the SRIE did not include information about time for primary prosthetic valve implantation or classification into early or late onset prosthetic IE, which makes it impossible to present or distinguish the etiology between early or late onset prosthetic IE. This would be of interest for the analysis of predisposition for specific bacteria or specific IE manifestations in this study. Comorbidities and underlying heart diseases such as degenerative structural heart disease and rheumatic heart disease (however relatively uncommon in this study and in Sweden in general), can contribute to important aspects of bacterial etiology, manifestations and mortality. Therefore, it should be taken into account that this study reflects the population of IE-patients from a large urban area, admitted to a University Hospital in Stockholm, Sweden and therefore may differ from other populations of IE-patients both nationally and globally. The SRIE does not include results from blood tests such as brain natriuretic peptide levels, which would have been interesting for the analysis especially in relation to in-hospital mortality. We were not able to assess how many days after initiating antibiotic treatment ECHO was conducted. Unfortunately, no follow-up information such as for example one-year mortality was included in the SRIE. In addition, it would be interesting to include in analysis, patients with IE with a more unusual etiology, such as infections with gram-negative bacteria and fungi; however, this was not possible in our study as there were very few cases with these infective agents. For this study, we did only include adult patients as no data from pediatric patients were available from the SRIE. To avoid selection bias, we included all patients admitted to the KUH from 2008 to 2017 that fulfilled the inclusion criteria.

## Conclusions

Significant associations were found between the incidence of certain bacterial species and specific IE manifestations detected by ECHO, such as aortic, mitral, tricuspid, or pulmonary valve vegetations, abscess formation, and CIED-associated IE. Associations were found between infections with certain bacterial species and more than one IE manifestation. Associations were also found between certain bacterial species and IE manifestations among patients with a history of IV drug abuse and those with prosthetic heart valves. It should be noted that the results from this study is based on a single-center cohort from an urban setting. Therefore, the results should be interpreted in relation to the population that we have studied.

Aortic valve vegetations were more common among male patients, but there were no differences between genders with respect to which bacterial species were the cause of IE. In current practice, ECHO is known to contribute to the diagnosis of IE and its lesions. However, the challenge for the future is to come closer to identifying which specific bacterial species are involved in the pathogenesis of IE, where ECHO might have a role. We believe that the results of the present study contribute with useful information about which pathogens can cause severe infections, on which valves, and that might require close follow-up with ECHO and surgical treatment. Further studies are needed to confirm the results in larger or different populations.

## Data Availability

The data that support the findings of this study were taken from the SRIE, but restrictions apply to the availability of these data, which were used under license for the current study, and so are not available publicly. Data are available from the corresponding author on reasonable request and with permission of the SRIE.

## Abbreviations

CI: confidence interval
CIED: cardiovascular implantable electronic device
CoNS: coagulase negative staphylococci
ECHO: echocardiography
HACEK: Haemophilus species, Aggregatibacter species, Cardiobacterium hominis, *Eikenella corrodens* and Kingella kingae
IE: infective endocarditis
IV: intravenous
KUH: Karolinska University Hospital
OR: odds ratio
PCR: polymerase chain reaction
PET-CT: positron emission tomography–computed tomography
SRIE: the Swedish national registry of infective endocarditis
TEE: transesophageal echocardiography
TTE: transthoracic echocardiography
